# Comparative Evaluation of the Digestive Enzyme Inhibition, Protein, and Starch Components of Ten *Macrotyloma uniflorum* (Lam.) Verdc. Accessions

**DOI:** 10.3390/plants14223483

**Published:** 2025-11-14

**Authors:** Queeneth A. Ogunniyi, Ada F. Molokwu, Abraham O. Nkumah, Abdullahi A. Adegoke, Olaniyi A. Oyatomi, Omonike O. Ogbole, Oluwatoyin A. Odeku, Joerg Fettke, Michael T. Abberton

**Affiliations:** 1 Department of Pharmacognosy, University of Ibadan, Ibadan 200132, Nigeria; queenethabiola@gmail.com (Q.A.O.); adafadey99@gmail.com (A.F.M.); abrahamnkumah32@yahoo.com (A.O.N.); adegokeabdullahi24@gmail.com (A.A.A.); 2 Genetic Resources Centre, International Institute of Tropical Agriculture (IITA), Ibadan 200001, Nigeria; o.oyatomi@cgiar.org (O.A.O.); m.abberton@cgiar.org (M.T.A.); 3 Department of Pharmaceutics and Industrial Pharmacy, University of Ibadan, Ibadan 200132, Nigeria; 4 Biopolymer Analytics, University of Potsdam, 14476 Golm, Germany

**Keywords:** *Macrotyloma uniflorum* (Lam.) Verdc, α-amylase, α-glucosidase, protein, resistant starch, amylose

## Abstract

Digestive enzymes play a crucial role in carbohydrate hydrolysis and subsequent glucose absorption, and their inhibition can contribute to improved glycemic regulation. Legumes, with their inherent enzyme-inhibitory properties, offer a natural approach for achieving this. In this study, accessions of *M. uniflorum* (Lam.) Verdc, an underutilized legume, were evaluated *in vitro* for their α-amylase and α-glucosidase inhibitory activities, as well as their protein, amylose, and resistant starch contents. The results revealed significant variation among the accessions. PI 174827 01 SD (IC_50_ = 23.29 ± 0.01 µg/mL) and PI 173901 01 SD (IC_50_ = 24.60 ± 0.01 µg/mL) demonstrated strong inhibition of α-amylase and α-glucosidase, respectively. Protein content ranged from 13.81 to 27.08%*w*/*w* d.w., with PI 180437 01 SD showing the highest percentage. Total starch content ranged from 27.48 to 54.70%*w*/*w* d.w., amylose from 27.05 to 48.13%*w*/*w* d.w., and resistant starch from 5.89% to 7.09%*w*/*w* d.w., with PI 174827 01 SD exhibiting both higher amylose and resistant starch contents. These findings suggest that *M. uniflorum* accessions possess enzyme-inhibitory and nutritional components that could be harnessed to develop functional foods, nutraceuticals, and pharmaceuticals for the management of diabetes and obesity.

## 1. Introduction

*Macrotyloma uniflorum* (Lam.) Verdc, or horse gram, is a less popular and underutilized legume in the Fabaceae family. It is widely grown in the southern part of Asia, Africa, Australia, and the West Indies by poor communities for food, fodder, and medicines [1,2,3]. Despite its important role in diets, it is often regarded as “food for the poor,” particularly in the Southern region of India [4]. *M. uniflorum* is a legume of significant nutritional and medicinal value. It is documented as a treasured source of protein and carbohydrates, and it also contains bioactive substances, including phytic acid, proteinase inhibitors, and phenolic acid, which have known biological effects [2,5]. Its consumption is associated with a low glycemic index, which promotes better glycemic control and may contribute to weight management [6].

*M. uniflorum* has been traditionally utilized in various forms, including soups, stews, and herbal remedies. Seed decoctions of horse gram are traditionally used as an astringent, diuretic, tonic, and for neuralgia, in addition to preventing various other disorders [7]. The seeds are also used as therapeutic agents for the treatment of cough, common cold, and fever, high cholesterol, spleen enlargement, and leucorrhea, and to aid in the dissolution of phosphate/calcium stones in the kidneys [8,9]. Although *M. uniflorum*, a nutrient-dense legume with recognized ethno-medicinal uses, holds significant potential to enhance human diet and health, its full benefits remain underexplored. Despite its nutritional value, *M. uniflorum* is restricted in production due to its anti-nutritional factors, such as phytates, oxalates, tannins, and trypsin inhibitors, which often interfere with nutrient bioavailability and protein and starch digestibility in the legume [10]. Additionally, oligosaccharides present in *M. uniflorum* contribute to flatulence and digestive discomfort upon ingestion [11]. However, many of the antinutrients that affect the digestibility of protein are sensitive to heat, suggesting that cooking the legume can enhance its protein digestibility [10]. Furthermore, the under-exploration of *M. uniflorum* in research, its limited awareness, and knowledge outside its traditional regions of consumption have contributed to its underutilization. Nevertheless, in developing countries, there is a growing focus on underutilized legumes as alternative protein sources to meet the increasing protein demands of expanding populations. As a result, interest in *M. uniflorum* is increasing due to its abundant content of proteins, carbohydrates, and bioactive metabolites, which offer a range of health advantages. The *M. uniflorum* seeds have been reported to contain potent anti-inflammatory, antimicrobial, antioxidant, antiadipogenic, and antilithiatic properties [2,12,13]. However, our area of particular interest is the digestive enzyme inhibitory activity of *M. uniflorum*, due to its potential role in managing disorders such as diabetes and obesity.

Enzyme inhibitors present in legumes are known to modulate the breakdown and absorption of carbohydrates and proteins, thereby influencing postprandial glucose levels and satiety [14]. Given the growing prevalence of diabetes and obesity, the identification of natural enzyme inhibitors in legumes like *M. uniflorum* is of considerable importance. Essentially, it is important to highlight that the digestive enzyme inhibitory properties and nutritional composition of *M. uniflorum* can vary significantly among different accessions due to genetic diversity, environmental factors, and cultivation practices. Understanding these variations is crucial for optimizing the incorporation of *M. uniflorum* into diets. Therefore, this study investigated the variation among ten accessions of *M. uniflorum,* specifically, their inhibitory activities against key digestive enzymes, and also evaluated their protein and starch components.

## 2. Materials and Methods

### 2.1. Seed Collection

Ten accessions of *M. uniflorum* (Lam.) Verdc stored at a temperature of about 5 °C were acquired from the Genetic Resource Centre (GRC) of the International Institute of Tropical Agriculture (IITA), Ibadan, Nigeria. Considering the aims of the International Treaty on Plant Genetic Resources for Food and Agriculture, the seed accessions were accompanied by a Standard Transfer of Agreement (SMTA) for the study.

### 2.2. Methanol Extraction for In Vitro Assays

Some seeds (20 seeds) from each of the accessions collected from the GRC were separately pulverized using a mortar and pestle. The pulverized samples were extracted with methanol in a macerating jar at room temperature for 72 h, with regular stirring, followed by filtration. Filtrates were concentrated *in vacuo* at 40 °C, and the extracts obtained were used for preliminary in vitro α-amylase and α-glucosidase inhibition assays.

### 2.3. Propagation of M. uniflorum Seeds

Seeds remaining from each accession were taken for propagation at the Pharmacy Garden of the University of Ibadan, Nigeria. A space in the garden was properly weeded, and ridges were made for ease of access and maintenance of the plants. The seeds were planted in loamy soil in the first week of July 2023 in nursery pots with 2–3 seeds per pot. Seedlings were later transplanted into loamy soil at the permanent site after 2 weeks. The plants were irrigated on days of no rainfall, and ridges were regularly weeded to maintain healthy plant growth. Seeds were harvested in September 2023 after 3 months of sowing. The seed accessions were dried, pulverized, and extracted in methanol, following the procedure for the methanol extraction of the GRC samples, and subjected to in vitro α-amylase and α-glucosidase inhibition assays for comparison of their activities against the GRC samples.

### 2.4. In Vitro Enzyme Inhibition Assays

#### 2.4.1. Reagents

Enzymes, substrates, pharmaceutical standards, and other commercially available reagents were procured from Sigma-Aldrich Chemical, Saint-Quentin-Fallavier, France and/or Merck KGaA, Darmstadt, Germany. These reagents include α-amylase from *Aspergillus oryzae* (10065), α-glucosidase from *Saccharomyces cerevisiae* (G5003), soluble starch from potato, *p*-Nitrophenyl-α-D-glucopyranoside (pNPG) (487506), 3,5-dinitrosalicylic acid (DNSA) (D0550), and Acarbose^®^ (PHR1253).

#### 2.4.2. *In Vitro* α-Amylase Inhibition

The assay was carried out using a modified method described by Ogbole et al. 2021 [15]. Methanol extracts of the *M. uniflorum* accessions were subjected to a two-fold serial dilution using an equal volume of 20 mM sodium phosphate buffer (pH 6.9), yielding six graded concentrations ranging from 1000 to 31.25 μg/mL. To each sample, 1 mL of α-amylase solution (2 U/mL) was introduced, followed by pre-incubation at room temperature for 30 min. The enzymatic reaction was initiated by the addition of 1 mL of potato starch solution (1.0% *w*/*v*) and subsequently incubated at room temperature for 5 min. The reaction was terminated by adding 1 mL of DNSA solution (96 mM 3,5-dinitrosalicylic acid and 5.3 M potassium sodium tartrate tetrahydrate in 2 M NaOH), followed by incubation at 80 °C for 15 min in a water bath. The reaction mixtures were then cooled and diluted with 9 mL of distilled water. Acarbose^®^ was used as the positive control, while the negative control represented 100% enzyme activity. A volume of 200 μL of each reaction mixture was dispensed into a microtiter plate, and absorbance was recorded at 540 nm using a Spectramax Gemini XS microplate reader (Sunnyvale, CA, USA).

#### 2.4.3. α-Glucosidase Inhibition

The assay was performed following the procedure reported by Ogbole et al., 2021 [15]. Similarly to the α-amylase assay, the methanol extracts of the *M. uniflorum* accessions were subjected to a two-fold serial dilution with the same volume of 0.1 M sodium phosphate buffer (pH 6.8), affording six graded concentrations ranging from 1000 to 31.25 μg/mL. To each sample, 100 µL of α-glucosidase solution (1 U/mL) was introduced and incubated for 10 min at room temperature. Afterwards, 50 µL of 3.0 mM p-nitrophenyl glucopyranoside (pNPG) solution was added to initiate the enzymatic reaction, followed by a 20 min incubation at room temperature. The reaction was subsequently terminated with 1.5 mL 0.1 M sodium carbonate (Na_2_CO_3_) solution. Acarbose^®^ was used as the positive control, while the negative control represented full enzyme activity (100%). Two hundred microliters (200 µL) of each reaction mixture were dispensed into a microtiter plate, and absorbance was taken at 405 nm using a Spectramax Gemini XS microplate reader (Sunnyvale, CA, USA).

The α-amylase and α-glucosidase inhibition assays were assessed in triplicate for each concentration and are presented as mean ± standard error of the mean (SEM) based on the three independent measurements. Percentage inhibition was calculated, and IC_50_ values for the extracts and acarbose were derived by graphing the percentage inhibition values against their respective concentrations.

### 2.5. Total Protein Content

About 50 mg of the pulverized cultivated seed accessions were extracted separately in 500 µL extraction buffer (1,4-Dithioerythritol (DTE), glycerin, 200 mM HEPES/2 mM EDTA/NaOH; pH 7.5, 200 mM Phenylmethylsulfonyl fluoride (PMSF), and ddH_2_O) and centrifuged at 4 °C, 14,000 rpm, for 15 min. The supernatant obtained was used to estimate the total protein content following the Bradford assay protocols and for sodium dodecyl sulphate–polyacrylamide gel electrophoretic (SDS-PAGE) separation of their proteins. Bovine serum albumin was used as the standard. The supernatant (5 µL) was diluted in 495 µL ddH_2_O, made up to 1000 µL with 500 µL Bradford reagent, and vortexed thoroughly. The mixture was kept at room temperature for approximately 5 min to facilitate colour development, and absorbance was taken at 595 nm on a UV-1900i UV–vis spectrophotometer (Shimadzu, Columbia, MO, USA) [16].

### 2.6. Electrophoretic Separation of Proteins

Proteins from the seed accessions were separated by 12% sodium dodecyl sulphate–polyacrylamide gel electrophoresis (SDS-PAGE) at 250 V and 30 mA. Following electrophoretic separation, gels were stained overnight with a commercially obtained Coomassie-blue staining solution (Roti Blue^®^-Roth, Karlsruhe, Germany). Subsequently, protein band intensities, visualized by Coomassie staining of SDS-PAGE gels, were quantified using ImageJ 1.51d image processing software. Protein bands of interest were excised from the gels and cut into smaller pieces. The gel pieces were repeatedly destained with a solution of 60% (*v*/*v*) 50 mM ammonium hydrogen carbonate and 40% (*v*/*v*) acetonitrile, incubated at 37 °C and 1200 rpm for 30 min in a thermoshaker until the stain was completely removed. The gel pieces were treated with 5–10 μL of 100% acetonitrile (*v*/*v*) for dehydration, followed by centrifugation to achieve complete dehydration, and digested with 20 μL of trypsin solution (0.02 μg trypsin/μL 50 mM NH_4_HCO_3_) and incubated at 37 °C for 20 h. The resulting peptides were analyzed by MALDI-TOF/MS using 0.35 µL peptide sample each with an equal volume of α-cyano-4-hydroxycinnamic acid (HCCA), which contained 3.5 mg HCCA dissolved in 1 mL 84% (*v*/*v*) acetonitrile, 13% (*v*/*v*) ethanol, and 3% (*v*/*v*) trifluoroacetic acid 0.1% (*v*/*v*), as a matrix. Mass spectra of peptides were acquired in positive ion reflector mode and subsequently matched against NCBI and Swissprot databases (accessed on 15 March 2024) through the MASCOT Peptide Mass Fingerprint and MASCOT MS/MS search tools (http://matrixscience.com) [17].

### 2.7. Total Starch Content

The total starch content was quantified enzymatically from 50 mg of the pulverized seed samples using the Megazyme Total Starch Assay Kit (Megazyme, Wicklow, Ireland), following the manufacturer’s protocol. Each sample was mixed with 1 mL of 80% *v*/*v* ethanol and shaken for 15 min at 80 °C in a thermoshaker. The mixture was centrifuged at 20,000× *g* for 10 min, and the sediment was rinsed with 1 mL of cold ddH_2_O and dried in a SpeedVac concentrator. In 0.5 mL of 0.2 M KOH, the sediment was resuspended and incubated at 95 °C for 1 h. Samples were neutralized with 88 µL of 1 M acetic acid and centrifuged at 14,000 rpm for 5 min. A 50 µL aliquot of the resulting supernatant was treated with 1 U of amyloglucosidase solution (R-Biopharm) and incubated at 55 °C for 1 h. To measure the starch concentration, 20 µL of each sample was made up to 1000 µL final volume with a buffer containing 200 mM Imidazole/HCL, pH 6.9, 3 mM MgCl_2_, 5.5 mM ATP, 2.5 mM NADP, and 1.7 U G6P-DH. Absorbance was recorded at 340 nm using a UV-1900i UV–vis spectrophotometer (Shimadzu, Columbia, MO, USA), with 0.5 U hexokinase serving as the catalyst.

### 2.8. Amylose Content

The amylose content of the seed accessions was measured using a small-scale iodine adsorption method [18] with slight modifications. In a 2 mL tube with a screw cap, 2 mg (accurate to 0.1 mg) of starch extracted from each seed accession was measured. Defatting was performed by adding 1 mL of 85% (*v*/*v*) methanol, followed by incubation at 65 °C for 1 h with periodic vortexing, and centrifugation at 13,000× *g* for 5 min. After centrifugation, the supernatant was removed, and the procedure for defatting was carried out a second time. The starch pellets were allowed to dry completely at 37 °C overnight and dissolved in UDMSO solution, comprising nine parts DMSO and one part 6 M urea, using a ratio of 1 mL UDMSO per 2 mg of starch. The samples were incubated at 85 °C for 2 h with periodic vortexing to achieve dissolution. To a 50 μL aliquot of starch-UDMSO solution, 20 μL of I_2_–KI reagent (2 mg iodine, 20 mg potassium iodide per mL of water) was added, and the final volume was brought to 1 mL with distilled water. The optical density of the samples was taken at 620 nm on a UV-1900i UV–vis spectrophotometer (Shimadzu, Columbia, MO, USA) [19].

### 2.9. Resistant Starch Content

The Megazyme^®^ Resistant Starch Assay Kit (Megazyme International Ireland Ltd. Wicklow, Ireland) was used to measure the resistant starch content of the isolated starch from the legumes following the AOAC Method 2002.02.

### 2.10. Data and Statistical Analyses

The IC_50_ values (expressed in µg/mL) of seed accessions from the Genetic Resource Centre (GRC) and the cultivated samples were compared using two-way Analysis of Variance (ANOVA) with GraphPad version 8.0.2. Total protein, starch, amylose, and resistant starch contents (expressed as % *w*/*w* d.w.) are reported as the mean ± Standard Error of Mean (SEM) of triplicate samples and duplicate samples for resistant starch. Differences in means were assessed using a one-way Analysis of Variance (ANOVA).

## 3. Results and Discussion

The identification of natural digestive enzyme inhibitors in legumes such as *M. uniflorum* holds significant potential for addressing the growing global concerns related to diabetes and obesity. One of the primary benefits of digestive enzyme inhibitors is their ability to regulate postprandial blood glucose levels [20,21]. Enzymes, including α-amylase and α-glucosidase, responsible for converting carbohydrates into simple sugars, play a pivotal role in glucose absorption into the bloodstream. The inhibition of these enzymes delays the digestion of carbohydrates, leading to slower and more controlled blood glucose levels [21]. In addition to carbohydrates, enzyme inhibitors may also influence the digestion of proteins and fats, ensuring that the body can better regulate the intake of these macronutrients [22]. This is especially beneficial for individuals with metabolic disorders, where efficient nutrient absorption plays a key role in maintaining a balanced metabolism and reducing insulin resistance.

An initial screening was conducted to evaluate the enzyme inhibitory effect of some seeds from each of the ten accessions of *M. uniflorum* collected from the GRC, where they were stored as part of a larger collection of crops. Crops in the GRC are stored to protect and conserve their genetic material, ensuring that valuable or rare crops such as *M. uniflorum* are available for future use. These crops are also stored for use in research to develop new crop varieties with beneficial traits such as increased yield, improved nutritional profile, and disease resistance. Afterwards, the remaining seeds of the accessions were cultivated, harvested, and subjected to the same assays. This allowed for a comparative analysis of the digestive enzyme inhibitory potential of *M. uniflorum* seed accessions from both sources and highlights the impact of storage and environmental factors on the bioactivity of the seeds.

**Figure 1:**
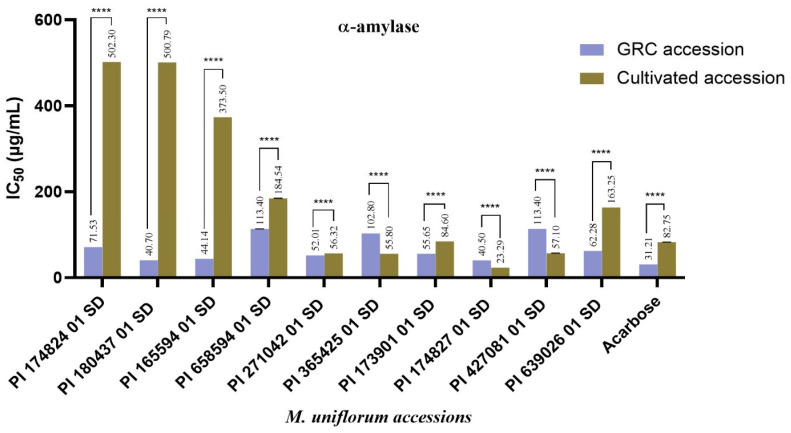
α-amylase inhibitory activity of *Macrotyloma uniflorum* accessions. Values are expressed as mean ± Standard Error of Mean (SEM) with *n* = 3. Means were compared between the GRC and cultivated accessions using Two-way ANOVA (α = 0.05). Data with **** are significantly different (*p* < 0.0001).

Overall, the digestive enzyme inhibitory effects of the seed accessions varied significantly between the GRC and cultivated seeds, as well as among accessions within each source. In comparison to the standard drug, Acarbose^®^ (IC_50_ = 82.75 ± 0.02 μg/mL), accessions PI 271042 01 SD (IC_50_ = 56.32 ± 0.01 μg/mL), PI 365425 01 SD (IC_50_ = 55.80 ± 0.01 μg/mL), PI 174827 01 SD (IC_50_ = 23.29 ± 0.01 μg/mL), and PI 427081 01 (IC_50_ = 57.10 ± 0.01 μg/mL), displayed enhanced inhibition of α-amylase, post-harvest while PI 180437 01 SD (IC_50_ = 40.7 ± 0.02 μg/mL), and PI 165594 01 (IC_50_ = 44.14 ± 0.01 μg/mL), which initially demonstrated better inhibition of α-amylase, displayed drastically reduced activity against the same enzyme with IC_50_ values of 500.79 ± 0.02 μg/mL and 373.50 ± 0.01 μg/mL, respectively (Figure 1). This enhanced inhibition exhibited by PI 271042 01 SD, PI 365425 01 SD, PI 174827 01 SD, and PI 427081 01 post-harvest may be attributed to the environment where they were grown and the quality of their secondary metabolites. Studies have demonstrated that plants of the same species can produce different concentrations and profiles of secondary metabolites when grown in different environments. This has been reported in soybeans, where the cultivated accessions of the bean were found to contain saponin type Aa, while saponin type AaBc was found to be prominent in the wild accessions of the soybean [23]. Also, in a study of seed isoflavones in 927 landraces and 241 cultivars of soybean, a higher total average isoflavone content was reported in landraces, and the least in one of the cultivars [24]. In contrast, the cultivated seed accessions, PI 174824 01 SD, PI 180437 01 SD, and PI 165594 01 SD, which displayed relatively low enzyme inhibition, especially against α-amylase despite the level of inhibition demonstrated by their GRC samples, may be linked to the cultivation process or environmental stressors such as temperature fluctuations, or nutrient limitations which may affect the synthesis of bioactive compounds, including those responsible for enzyme inhibition during cultivation [25].

**Figure 2:**
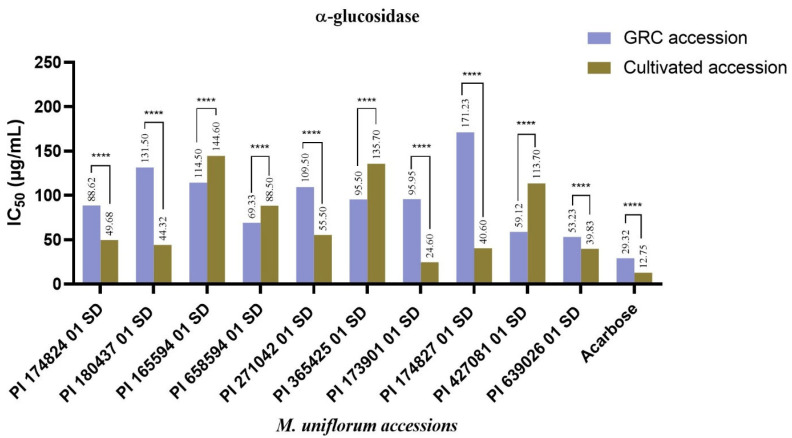
α-glucosidase inhibitory activity of *Macrotyloma uniflorum* accessions. Values are expressed as mean ± Standard Error of Mean (SEM) with *n* = 3. Means were compared between the GRC and cultivated accessions using Two-way ANOVA (α = 0.05). Data with **** are significantly different (*p* < 0.0001).

Similarly, while the cultivated seed accessions PI 165594 01 SD, PI 658594 01 SD, PI 365425 01 SD, and PI 427081 01 SD demonstrated reduced activity against α-glucosidase when compared to their GRC samples, other accessions, particularly PI 173901 01 SD; PI 174827 01 SD, which inhibited α-amylase the most; and PI 639026 01 SD, inhibited α-glucosidase better post-harvest (Figure 2). The identified differences in the enzyme inhibition of some seed accessions of *M. uniflorum* collected from the GRC and the cultivated samples may be influenced by storage and environmental factors. Seeds obtained from the GCR, IITA, Ibadan, Nigeria, are often stored at a temperature of about 5 °C for the preservation of their genetic integrity and other essential components. Their storage period in the GRC also varies, and this could lead to degradation or changes in the composition of their bioactive metabolites over time. Additionally, during storage, these seeds may experience physiological stress owing to the buildup of reactive oxygen species (ROS), which can oxidize organic compounds and cause irreversible damage to key bioactive components in the seeds [26,27]. This may explain why some of the seed accessions obtained from the GRC exhibited reduced activity, particularly against α-glucosidase.

Among the seeds collected from the GRC, PI 174827 01 SD (IC_50_ = 40.5 ± 0.01 μg/mL) demonstrated the best α-amylase inhibitory effect comparable to PI 180437 01 SD (IC_50_ = 40.7 ± 0.02 μg/mL) and PI 165594 01 SD (IC_50_ = 44.14 ± 0.01 μg/mL) (Figure 1), while PI 427081 01 SD (IC_50_ = 53.23 ± 0.02 μg/mL) and PI 427081 01 SD (IC_50_ = 59.12 ± 0.03 μg/mL) exhibited the best inhibition of α-glucosidase (Figure 2). Among the cultivated accessions, PI 174827 01 SD (IC_50_ = 23.29 ± 0.01 μg/mL), PI 365425 01 SD (IC_50_ = 55.80 ± 0.01 μg/mL), PI 271042 01 SD (IC_50_ = 56.32 ± 0.01 μg/mL), and PI 427081 01 SD (IC_50_ = 57.10 ± 0.01 μg/mL) strongly inhibited α-amylase better than Acarbose^®^ (IC_50_ = 82.75 ± 0.02 μg/mL) (Figure 1). In contrast, PI 173901 01 (IC_50_ = 24.60 ± 0.01 μg/mL), PI 639026 01 (IC_50_ = 39.83 ± 0.01 μg/mL), and PI 174827 01 (IC_50_ = 40.60 ± 0.01 μg/mL) displayed better inhibition of α-glucosidase (Figure 2). It is worthy of note that the accession PI 174827 01 SD, which is one of the three accessions identified to possess a good inhibition of α-glucosidase among the cultivated accessions, demonstrated the highest α-amylase inhibition among the GRC samples, and also inhibited the same enzyme the most among the cultivated accessions. This suggests that PI 174827 01 SD is particularly significant because it exhibited a strong inhibition of both α-amylase and α-glucosidase, which may have potential applications in managing diabetes by influencing carbohydrate digestion and glucose absorption. The variation observed in the inhibition of α-amylase and α-glucosidase by the seed accessions of *M. uniflorum* is not surprising, given the inherent genetic differences between the accessions. Each accession possesses a unique genetic makeup, which can lead to variations in the expression of the genes responsible for producing bioactive compounds. As a result, the genetic diversity among the accessions influences not only the quantity but also the types of bioactive components present in the seed accessions. Secondary metabolites in *M. uniflorum* accessions may contribute to the inhibition of α-amylase and α-glucosidase. To corroborate this, a study reported that *M. uniflorum* α-amylase inhibitor (MUAI) isolated from its seeds inhibited α-amylase obtained from mouse pancreas and human saliva, non-competitively. Additionally, the MUAI reduced serum glucose levels in diabetic mice, with minimal pathological changes observed in the mice treated with MUAI compared to the diabetic control [28].

Focusing on the cultivated seed accessions of *M. uniflorum*, the total protein, starch, amylose, and resistant starch contents and the protein profile using SDS-PAGE were evaluated. The investigation of these components across the different seed accessions further highlighted the genetic diversity within the species (Table 1).

The total protein content differed significantly among the accessions, ranging from 13.81 ± 0.46%*w*/*w* d.w. in PI 271042 01 SD to 27.08 ± 0.66%*w*/*w* d.w. in PI 180437 01 SD. The total starch, amylose, and resistant starch contents also, significantly varied among the seed accessions with PI 639026 01 SD (54.70 ± 4.51%*w*/*w* d.w.) exhibiting the highest total starch content while PI 174827 01 SD (48.13 ± 0.26%*w*/*w* d.w.) had the most amylose and PI 658594 01 SD (7.09 ± 0.00%*w*/*w* d.w.) showed the highest resistant starch. Notably, accession PI 174827 01 SD, which displayed the best α-amylase inhibition, also had the most amylose content and a relatively high amount of resistant starch. Amylose, a slower-digesting component of starch, is known to be less readily broken down by α-amylase. This is because it reduces the accessibility of α-amylase to the α-1,4 glycosidic bonds due to its linear structure and tight helical formation [29,30]. Regardless of this, the inhibitory effect of PI 174827 01 SD can not be attributed to its high amylose content and resistant starch content since the extracts would not have contained these macromolecules. Additionally, the genetic profile of PI 174827 01 SD may favour the production of both high levels of amylose and resistant starch, as well as the synthesis of compounds that inhibit α-amylase activity. In contrast, accession PI 180437 01 SD, which demonstrated a relatively low inhibition of α-amylase (Figure 1), low levels of amylose (27.05 ± 0.51%*w*/*w* d.w.), and resistant starch (5.89 ± 0.00%*w*/*w* d.w.), had the highest protein content (Table 1). This observation in protein, amylose, and resistant starch contents, as well as the reduced α-amylase inhibition in PI 180437 01 SD, may be influenced by environmental factors during cultivation. Conditions such as soil type, water availability, and temperature can significantly affect the metabolic pathways of the plant, including those involved in the biosynthesis of phenolic compounds, flavonoids, and saponins, which are known to contribute to enzyme inhibition. Conversely, optimal conditions may favour protein synthesis but reduce the production of these secondary metabolites. However, since all the accessions were cultivated under the same environmental conditions, these differences most likely reflect intrinsic genetic differences among accessions in their capacity to synthesize these bioactive compounds. Additionally, genetic expression may vary under different conditions, with the cultivation environment favouring protein production over starch synthesis. Compared to the accessions with the highest total protein, starch, amylose, and resistant starch, a good amount of these components was also found in some other accessions. Furthermore, while a positive correlation was observed between the total starch, amylose, and resistant starch contents of PI 658594 01 SD, PI 174827 01 SD, and PI 639026 01 SD, a contrasting trend was observed in some other accessions such as PI 174824 01 SD which exhibited relatively high amylose and resistant starch despite having the least total starch content, and PI 180437 01 SD which had a high total starch content but the lowest amount of amylose and resistant starch (Table 1). This implies that the amylose and resistant starch content of the accessions may not be entirely dependent on the quantity of their starch but on other factors, including their genetic makeup, starch composition, such as the ratio of amylose to amylopectin, and their starch granule structure [31,32]. Isolated proteins from each accession of *M. uniflorum* were separated on the gel based on their molecular weight using the 12% SDS-PAGE. Most of the proteins present in the accessions were found between 11 and 100 kDa. MALDI–TOF/MS was employed to identify individual proteins by searching against proteins in the SwissProt database.

**Figure 3:**
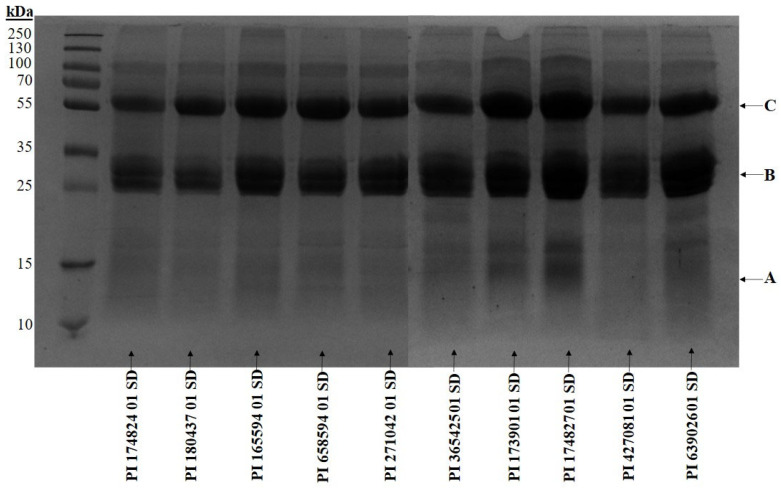
The 12% SDS-PAGE protein profile of *Macrotyloma uniflorum* accessions.

The protein profiles showed a similar pattern among all the accessions, with defensin (A), lectin (B), and protein kinase (C) identified as having molecular weights of 11 kDa, 29 kDa, and 65 kDa, respectively (Figure 3). Lectin was observed to be prominent in all the accessions of *M. uniflorum,* including PI 173901 01 SD and PI 174827 01 SD, which demonstrated the strongest inhibition of α-glucosidase and α-amylase, respectively, as well as protein kinase, while defensin was predominant in accessions PI 173901 01 SD and PI 174827 01 SD only.

Among the identified proteins, lectins, which are carbohydrate-binding proteins, possess the ability to inhibit digestive enzymes such as α-amylase and α-glucosidase. Supporting this, chickpea (*Cicer arietinum* L.) lectin has been shown to significantly inhibit both α-glucosidase and α-amylase in a concentration-dependent way [33]. However, the inhibitory effects observed in accessions PI 173901 01 SD and PI 174827 01 SD against α-glucosidase and α-amylase, respectively, cannot be linked to their lectin content, as lectins are typically not efficiently extracted with methanol. The prominence of lectins in these accessions may instead reflect an evolutionary adaptation that provides both defence against herbivores and influence carbohydrate metabolism. Plants frequently increase lectin expression as a defence mechanism against herbivores that feed on them [34,35]. Additionally, lectins help regulate carbohydrate digestion in the plant by interacting with specific carbohydrates, potentially influencing nutrient processing and storage [36,37]. This could also explain why some of the accessions were found to have high levels of protein, amylose, and resistant starch.

*M. uniflorum* may be considered a valuable resource for the development of functional foods, nutraceuticals, and pharmaceuticals to support glucose regulation and overall metabolic health in diabetic individuals. However, further investigations are necessary to explore the bioactive components of the legume accessions responsible for the inhibition of α-amylase and α-glucosidase.

## 4. Conclusions

The different accessions of *M. uniflorum* (Lam.) Verdc revealed notable variations in key traits, including protein, starch, amylose, and resistant starch contents, as well as their enzyme-inhibitory effect. These differences highlight the genetic diversity present within the species. Accession PI 174827 01 SD, which exhibited high α-amylase inhibition alongside high amylose and resistant starch content, suggests the presence of bioactive compounds that could be utilized in managing diabetes and obesity in the species. This accession could be selected for use in developing functional foods aimed at regulating postprandial glucose levels. In addition, accessions with promising nutritional traits may serve as valuable genetic resources for breeding programmes focused on improving the nutritional quality of underutilized legumes.

Although *M. uniflorum* is traditionally consumed in cooked forms such as stews and soups, the present findings were obtained from raw seed extracts. Therefore, the reported enzyme inhibitory activities represent potential bioactivity in uncooked seeds. Further studies using cooked or processed samples are needed to evaluate the stability and relevance of its constituents. Overall, this work provides foundational data for understanding the nutritional and functional diversity of *M. uniflorum* and may guide the selection of accessions with desirable properties for future breeding, nutritional enhancement, and functional food development.

## Figures and Tables

**Table 1:** Total protein, amylose, starch, and resistant starch contents of *Macrotyloma uniflorum* accessions.

Accession	Total Protein (%*w*/*w* d.w)	Total Starch (%*w*/*w* d.w.)	Resistant Starch (%*w*/*w* d.w.)	Amylose Content (%*w*/*w* d.w.)
PI 174824 01 SD	24.13 ± 0.98 ^a^	27.48 ± 1.89 ^f^	6.99 ± 0.00 ^ab^	36.07 ± 0.68 ^e^
PI 180437 01 SD	27.08 ± 0.66 ^a^	38.83 ± 5.56 ^d^	5.89 ± 0.00 ^d^	27.05 ± 0.51 ^f^
PI 165594 01 SD	14.90 ± 0.25 ^c^	38.17 ± 2.81 ^d^	7.04 ± 0.00 ^a^	34.57 ± 0.28 ^e^
PI 658594 01 SD	26.64 ± 0.53 ^a^	51.23 ± 2.99 ^b^	7.09 ± 0.00 ^a^	38.28 ± 0.58 ^d^
PI 271042 01 SD	13.81 ± 0.46 ^c^	33.60 ± 4.94 ^e^	6.03 ± 0.00 ^d^	35.12 ± 0.30 ^e^
PI 365425 01 SD	13.85 ± 0.50 ^c^	34.01 ± 6.92 ^e^	6.65 ± 0.00 ^b^	42.76 ± 1.55 ^c^
PI 173901 01 SD	18.69 ± 0.73 ^b^	42.84 ± 5.55 ^c^	6.31 ± 0.01 ^c^	38.68 ± 0.55 ^d^
PI 174827 01 SD	19.19 ± 0.28 ^b^	40.05 ± 5.09 ^cd^	6.97 ± 0.00 ^ab^	48.13 ± 0.26 ^a^
PI 427081 01 SD	14.67 ± 0.31 ^c^	42.08 ± 4.02 ^c^	6.49 ± 0.01 ^c^	46.24 ± 0.74 ^b^
PI 639026 01 SD	21.19 ± 0.80 ^b^	54.70 ± 4.51 ^a^	6.79 ± 0.01 ^b^	40.49 ± 0.93 ^d^

Values are expressed as mean ± Standard Error of Mean (SEM) with *n* = 3 for total protein, starch, and amylose contents and *n* = 2 for resistant starch content. Means were compared between the accessions using Ordinary one-way ANOVA. Data with the different superscript letters in the same column are significantly different (*p* < 0.0001). d.w.: dry weight.

## Data Availability

Data can be made available on request.
